# The cell-cycle choreography of H3 variants shapes the genome

**DOI:** 10.1016/j.molcel.2023.08.030

**Published:** 2023-11-02

**Authors:** Kamila Delaney, Nicole Weiss, Geneviève Almouzni

**Affiliations:** 1 Institut Curie, PSL Research University, CNRS, Sorbonne Université, Nuclear Dynamics Unit, Equipe Labellisée Ligue contre le Cancer, 26 rue d’Ulm, 75005 Paris, France

**Keywords:** H3 histone variants, chromatin dynamics, DNA replication

## Abstract

Histone variants provide versatility in the basic unit of chromatin, helping to define dynamic landscapes and cell fates. Maintaining genome integrity is paramount for the cell, and it is intimately linked with chromatin dynamics, assembly, and disassembly during DNA transactions such as replication, repair, recombination, and transcription. In this review, we focus on the family of H3 variants and their dynamics in space and time during the cell cycle. We review the distinct H3 variants’ specific features along with their escort partners, the histone chaperones, compiled across different species to discuss their distinct importance considering evolution. We place H3 dynamics at different times during the cell cycle with the possible consequences for genome stability. Finally, we examine how their mutation and alteration impact disease. The emerging picture stresses key parameters in H3 dynamics to reflect on how when they are perturbed, they become a source of stress for genome integrity.

## Teaser

While the canonical core histones comprise typical nucleosomes, histone variants provide unique features linked to a variety of chromatin and DNA transactions. Delaney, Weiss, and Almouzni review the importance of the dynamics of histone H3 variants and how mutations of these variants or perturbations in their dynamics can impact genome integrity and disease.

## Introduction

The dynamic exchanges of histone variants, post-translational modifications (PTMs), and DNA methylation, along with the interactions of specific regulatory factors, non-coding RNA, and architectural components, create the diverse landscapes corresponding to distinct cell fates and identities.^1^ The orchestration of these dynamics should consider all the challenges associated with various DNA transactions important for life. Thus, throughout the cell cycle, coordinating chromatin dynamics and maintaining DNA integrity are intimately linked when DNA replicates and reassembles into chromatin, when it is damaged and repaired, and when it is unwound for transcription. In each of these events, chromatin undergoes disassembly and reassembly and ensures genome protection. Indeed, inadequate amounts of histones have been associated with genome instability in various systems.^2^^,^^3^ Furthermore, the choice of distinct variants marking different regions of the genome is also important to consider in maintaining genome stability. Here, we will focus on the histone H3 family, comprising mainly H3.1, H3.2, H3.3, and centromeric protein A (CENP-A), which themselves have been linked to genome stability. For instance, loss of H3.3 or CENP-A exhibits chromosome instability (CIN) and aneuploidy.^4^^,^^5^ Given the links of the variants with cell cycle and genome instability, exploring H3 dynamics throughout the cell cycle is essential to understand their roles in the various stress responses in cells.

This review aims to discuss the orchestration of H3 variants’ dynamics with their associated chaperones in chromatin throughout time and space, with particular emphasis on their roles in genome stability. We first give an overview of the different H3 variants and how they come in and out of chromatin during the cell cycle. Next, we present their general genome-wide enrichment patterns. We also introduce how partnerships between chaperones and distinct variants contribute to these selective enrichments. Moving to the evolutionary scale, we discuss the emergence of new chaperones alongside important H3.3 functions. We detail how H3 variant patterns are deposited in S-phase and re-established following fork passage. Lastly, we discuss the function of H3.3 in humans through the lens of disease, highlighting oncohistone mutations. Altogether, we hope to emphasize the importance of H3 variants while underscoring their imperative role in genome integrity.

## Who: The players and their dynamics

**Figure 1:**
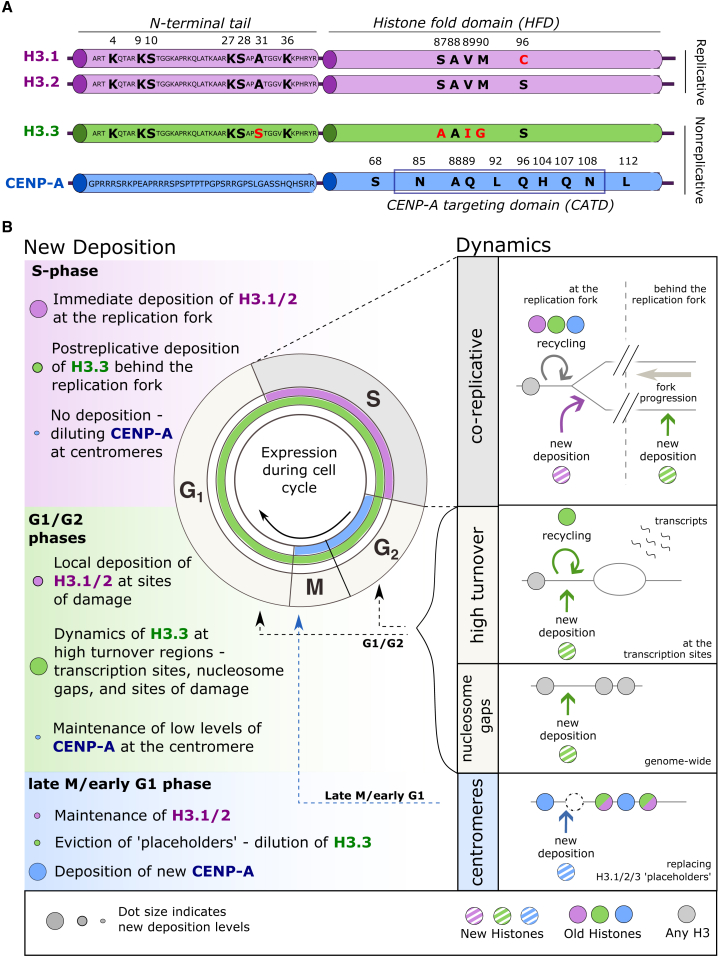
Histone H3 variants in mammals, their cell-cycle expression, and general dynamics (A) The scheme displays the main variants in the H3 family, i.e., replicative H3.1 and H3.2 and non-replicative H3.3 and CENP-A. In the two domains, difference in amino acid sequence is indicated in red and bold. H3.1/2 share 96% identity with H3.3, but these variants share only 50% identity with CENP-A. While we show the main four members of the H3 family, there are numerous additional members.^6^ (B) The dynamics of H3 variants include regulation of their expression throughout the cell cycle (inset), time for their new deposition, and recycling. The replicative variants H3.1/2 show the peak of expression in S-phase, and the newly synthesized H3 is deposited mainly during replication. In contrast, the non-replicative variants H3.3 and CENP-A do not show a peak in S-phase. H3.3 is steadily expressed throughout the cell cycle and can be deposited at high turnover regions. CENP-A expression peaks in G2/M, and deposition of the newly synthesized CENP-A occurs in late M/early G1.

In the H3 histone family, we will consider four main proteins, H3.1, H3.2, H3.3, and CENP-A (Figure 1A). Other members exist^6^ and more have been identified.^7^ The first H3 variant identification relied on distinct migration properties on Triton acid urea gels.^8^ Not long after, expression timing differences between the variants observed in Chinese hamster ovary (CHO) cells enabled the classification of replicative and non-replicative variants.^9^ The high S-phase expression of certain variants corresponds to replicative histones, mainly H3.1 and H3.2. Non-replicative histones, which do not peak in S-phase, comprise H3.3, which is expressed steadily throughout the cell cycle and in quiescent cells.^6^ The peak of CENP-A expression in G2/M also placed it in the non-replicative group.^10^^,^^11^ These distinct expression profiles (Figure 1B, inset circle) provided the first functional differences between the H3 variants.

Amino acid composition and their variation across the H3 family is also revealing. Indeed, to quote David Allis, “every amino-acid matters.”^12^ In mammals, H3.1 and H3.2 are 99% identical and share 96% sequence identity with H3.3, differing by 5 or 4 residues, respectively (Figure 1A). Three of these changes fall in the histone fold domain (HFD) over amino acids 87–90: SAVM motif in H3.1/2 is replaced with AAIG in H3.3 (Figure 1A). Beyond this, a last difference renders these variants functionally non-interchangeable as we will discuss later. Here, position 31 in the N-terminal tail is alanine in H3.1/2 and serine in H3.3. Serine 31 (S31), a residue that can be reversibly phosphorylated, influences biology in a manner where H3.1/2 cannot compensate. This importance includes a need for the rapid transcriptional activation in embryonic stem (ES) cells,^13^ macrophage activation,^14^ and an essential role in development during gastrulation in *Xenopus*.^15^ CENP-A, the centromeric histone variant, in contrast to the rest of the H3 family, shares less than 50% homology with the other variants (Figure 1A). This divergence has been attributed to its rapid evolution along with the evolution of centromeres.^16^ Indeed, the specific deposition at centromeres relies on a CENP-A-targeting domain (CATD)^17^^,^^18^^,^^19^ (Figure 1A). The differences among the H3 family concern not only expression and sequence variations but also their deposition mode.

Indeed, the deposition of newly synthesized replicative H3.1 and H3.2 proceeds in a DNA synthesis-coupled (DSC) manner,^20^^,^^21^ as opposed to the distinct DNA synthesis-independent (DSI) pathways used by H3.3^21^^,^^22^ and CENP-A.^17^^,^^18^ However, histone dynamics involve both “new” histone deposition and “old” histone recycling to maintain and/or change states. Replicative H3.1/2, following its peak expression at G1/S, provides most of the material for new H3 deposition during S-phase, coupled to replication, and operates genome-wide (Figure 1B, top) (detailed in section H3 dynamics at the replication fork). When H3.1/2 is not expressed, new deposition relies significantly on H3.3 (Figure 1B, middle), for example, out of S-phase or in long-lived cells and in quiescence. Notably, both H3.3 recycling and new deposition occur during transcription, when nucleosomes are evicted at sites of active transcription,^23^ and other high turnover regions such as sites of repair. Finally, following its peak of expression at G2/M phase,^10^ new CENP-A incorporation starts during late mitosis telophase/G1 phase (Figure 1B, bottom),^24^ although this timing may differ across species.^25^ While the variants demonstrate clear distinction in expression, sequence, and deposition mode and timing, they also show distinct genomic enrichment.

## Where: Genomic landscapes of H3 variants and chromatin landmarks

**Figure 2:**
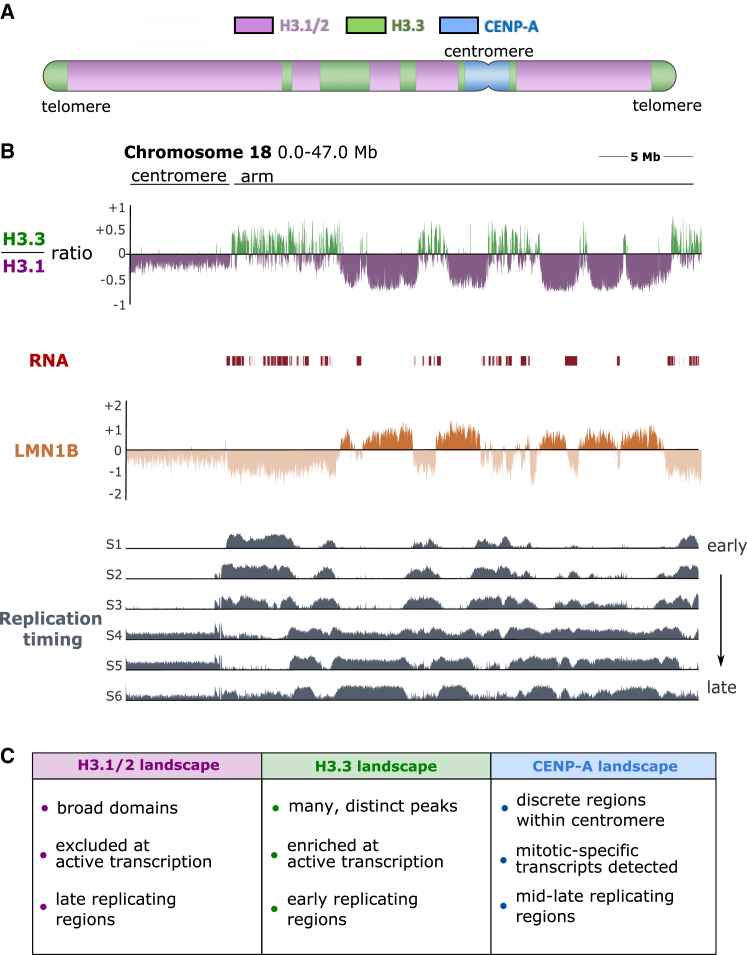
General H3 variant enrichment across the genome (A) Scheme H3.1 and H3.3 enrichment across a section of chromosome 18 (chr18), indicating H3.1 broad enrichment across the chromosome; H3.3 at discrete peaks corresponding to transcription sites, promoter regions or gaps, as well as at heterochromatic regions (pericentromeres, telomeres). CENP-A enrichment, in contrast, localizes precisely at the centromere. (B) H3.1 and H3.3 enrichment across ∼50 Mb segment of chr18; plots are shown for the ratio of H3.3/H3.1 enrichment,^26^ nascent RNA mapping,^27^ Lamin B enrichment,^29^ and replication timing of indicating genomic regions.^28^ (C) Summary of the characteristics for each variant—H3.1/2, H3.3 (based on above citations), and CENP-A.^35^

The different H3 variants harbor distinct genome-wide distributions in cycling cells.^26^ Replicative H3.1/2 enrichment occurs in large blocks or domains (Figures 2A and 2B, purple), mainly non-transcribed^27^ and late-replicating regions,^28^ often associated with nuclear lamina^29^ and enriched in heterochromatin marks such as H3K9me3. In contrast, H3.3 enrichment shows discrete peaks^26^ (Figures 2A and 2B, green) often coinciding with active transcription^27^ and early-replicating regions,^28^ also enriched in PTMs like H3K4me3 and H3K36me3, considered marks of active transcription. In addition, particular heterochromatin regions show H3.3 presence, like telomeres, or repetitive regions in the genome, as reported in mouse cells.^30^^,^^31^ Whether or not the roles of H3.3 in euchromatic and heterochromatic regions are the same remains an open question. However, it is tempting to hypothesize that RNA synthesis may be a trigger for H3.3 deposition in heterochromatic regions.^32^ Finally, CENP-A typically marks centromeres (Figure 2A, blue), one per chromosome with a strong enrichment extending over megabase-long repetitive domains as shown in chromatin immunoprecipitation (ChIP) experiments.^33^ In mammals, these centromeric regions correspond to mid- to late-replicating domains,^28^ and transcription detected at the centromere promotes CENP-A deposition and thereby favors centromere maintenance.^34^ Recently, thanks to long-read sequencing and the Telomere-to-Telomere (T2T) consortium effort,^35^ a refined mapping of CENP-A at α-satellite higher-order repeats showed a negative correlation with CpG methylation.^36^ These megabase-long CENP-A enrichment domains segmented into discrete regions within centromeres,^35^ which could reflect the interspersed organization with H3.1 and H3.3 found with combined chromatin fiber and SNAP-tag analysis.^37^ It is thus important to consider all variants in combination and their respective roles in the maintenance of centromeric architecture and function. Taken together, the genomic distributions of the H3 variants correspond to functional landmarks. It is thus important to understand how defined histone chaperones contribute to distinct deposition pathways and channel the variants to their destination.

## H3 chaperones—the escorts showing the way

**Figure 3:**
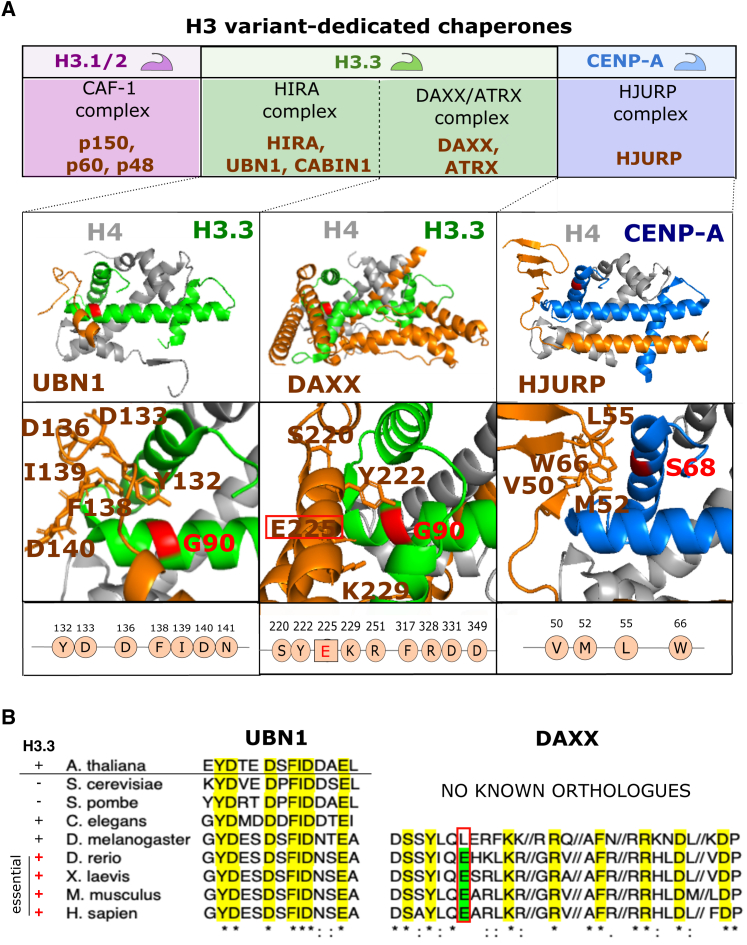
H3 variant-dedicated chaperones for new deposition (A) Top row indicates the known specific H3 chaperone complexes with their associated subunits; in the mid-row, the structural models for histone dimers H3-H4 and the corresponding interacting subunit for H3.3 and CENP-A: UBN1,^38^ DAXX,^39^ and HJURP^40^; there is currently no structural information for CAF-1 in complex with H3.1/2. The bottom row provides a zoom on the interacting residues between the chaperone subunit and H3 variant. Important residues in the chaperone are highlighted in brown, and the key residue in H3.3 or CENP-A is in red. The corresponding amino acid stretch in the chaperones is indicated below. (B) Evolutionary conservation of interacting residues of UBN1 (left) and DAXX (right) across selected species. Of note, DAXX is only found starting in *Drosophila*, and it shows a different amino acid (L) instead of E that is conserved in the species indicated underneath.

Histone chaperones play critical roles in escorting the H3 variants to maintain chromatin integrity and preserve nucleosomal landscapes. Here, we will focus on the main class of chaperones dedicated to specific H3 variants deposition onto DNA. We consider four key protein complexes: chromatin assembly factor 1 (CAF-1) complex, histone regulator A (HIRA) complex, death domain-associated and alpha-thalassemia/mental retardation, X-linked protein (DAXX/ATRX) complex, and Holliday junction recognition protein (HJURP) complex (Figure 3A, top row).

CAF-1, identified based on its unique capacity to promote chromatin assembly coupled to DNA synthesis,^41^ comprises three subunits—p150, p60, and p48^42^ (Figure 3A, top left). Found in complex with newly synthesized H3.1/2, CAF-1 does ensure the deposition of the replicative variants in a DSC manner^21^ during S-phase at replication sites^43^ and outside of S-phase at DNA repair sites.^44^

In contrast, the non-replicative H3.3 associated with the HIRA complex^21^ uses a DSI deposition pathway. In fact, there are two distinct complexes—HIRA^21^^,^^45^^,^^38^ and ATRX/DAXX^46^^,^^47^^,^^48^ (Figure 3A). The distinction between the nearly identical variants takes advantage of the specific recognition of the AAIG motif in H3.3 over SAVM in H3.1/2 (Figure 1A). The HIRA complex associates with the new deposition of H3.3 into euchromatic regions of chromosome arms.^47^^,^^49^ A HIRA trimer forms a complex with two additional subunits—ubinuclein 1 (UBN1) and calcineurin-binding protein 1 (CABIN1)^50^^,^^51^^,^^52^ (Figure 3A). While the exact function of CABIN1 is not fully understood, UBN1 is responsible for the direct binding of H3.3, an interaction depending on ∼7 evolutionarily conserved amino acids^53^ (Figures 3A and 3B). In mammals, UBN2^50^ can also interact with HIRA, although its role is not fully characterized. While HIRA trimerization and the UBN1 subunit are essential for new deposition of H3.3,^52^ they are dispensable for H3.3 recycling during transcription.^54^ Indeed, the HIRA complex also promotes recycling of H3.3 during transcription where it requires interaction with the chaperone anti-silencing function protein 1 (ASF-1).

Within the ATRX/DAXX complex, DAXX binds H3.3 directly.^39^ H3.3 depends on the DAXX/ATRX complex for incorporation into heterochromatin regions, i.e., telomeres, pericentric chromatin, and certain types of retroviral repeats.^46^^,^^47^^,^^55^^,^^56^^,^^57^ Since recent work showed that DAXX can bind SETDB1 and SUV39H1/2 to promote H3K9 methylation of its associated H3.3-H4 dimers, imposing this repressive mark prior/or at the time of histone deposition could aid heterochromatin maintenance/stability.^58^ This observation is in line with an importance of H3.3 and K9me3^59^ for silencing some heterochromatic regions, especially in embryonic and non-terminally differentiated cells.^30^^,^^56^^,^^60^^,^^61^^,^^62^

Finally, the centromeric variant CENP-A preferentially interacts with the HJURP chaperone^17^^,^^18^ (Figures 3A and 3B), which ensures both CENP-A recycling^63^ and deposition at centromeres. Following the identification of HJURP,^17^^,^^18^ analysis of the complex^19^ identified amino acids in the HFD of CENP-A, critical for its centromeric deposition—the so-called CATD^18^^,^^19^ (Figure 1A). Later, the structure of HJURP bound to a CENP-A:H4 heterodimer further shed light on an additional residue within the histone fold, S68, which influences the specificity of this interaction^40^ (Figure 3A).

How H3 variants and their dedicated chaperones evolved and how their perturbation impacts the life of organisms are informative. Indeed, the appearance of separate variants during evolution is linked with the increasing complexity of the organism.^6^ While H3 as a single variant in yeast is essential,^64^ it can use both DSC and DSI pathways. The appearance of H3.3 as a separate variant in the species *Tetrahymena*, worms, and flies does not make it essential, while it is required in zebrafish, frogs, and mice (Figure 3B). The dispensability of H3.3 in some organisms may actually reflect leaky expression of H3 variants combined with promiscuous interactions with chaperones. Thus, a strong requirement for variants in other species may relate to constrained partnerships with distinct chaperones. We compare key histone-interacting proteins within chaperone complexes across a series of species (Figure 3B). Within the HIRA complex, UBN1 directly interacts with H3.3^38^ (Figure 3A). Even though UBN1 itself is not a conserved protein, the Hpc2-related domain (HDR) responsible for specific H3.3 recognition is conserved among the species examined (Figure 3B, left). Each has a UBN1 ortholog and shows perfect conservation of the 7 critical residues key for H3.3 binding^38^ (Figure 3B, left). This observation supports the view that the HIRA-mediated non-replicative H3 deposition pathway is conserved from yeast to human. Analysis of DAXX, the H3.3-binding protein within DAXX/ATRX complex, tells a different story. Unlike UBN1, a DAXX ortholog is only found in flies, zebrafish, frog, mouse, and human, in the species examined (Figure 3B, right), although a putative yeast ortholog, Rtt106p, has been identified with significant sequence differences.^65^ While the overall protein conservation is not high from fly to human, important H3.3-interacting residues within the histone binding domain are conserved.^48^ Of these, residue E225, responsible for specific binding with G90 (present on H3.3) over M90 (present in H3.1/2), is conserved in all species noted except flies. Remarkably, the emergence of the E225 residue in DAXX coincides with H3.3 becoming essential (Figure 3B), which provides an argument that E225 is critical for survival in the corresponding species; however, as this is an open question, further exploration is required to investigate the essentiality of this residue. Further investigation may be warranted into DAXX-mediated H3.3 depositions for which other pathways in these species cannot compensate.

Taken together, histone-chaperone associations constitute an important axis shaping chromatin assembly. We now examine the interplay between the variants and their chaperones operating during and after DNA replication.

## H3 dynamics at the replication fork

**Figure 4:**
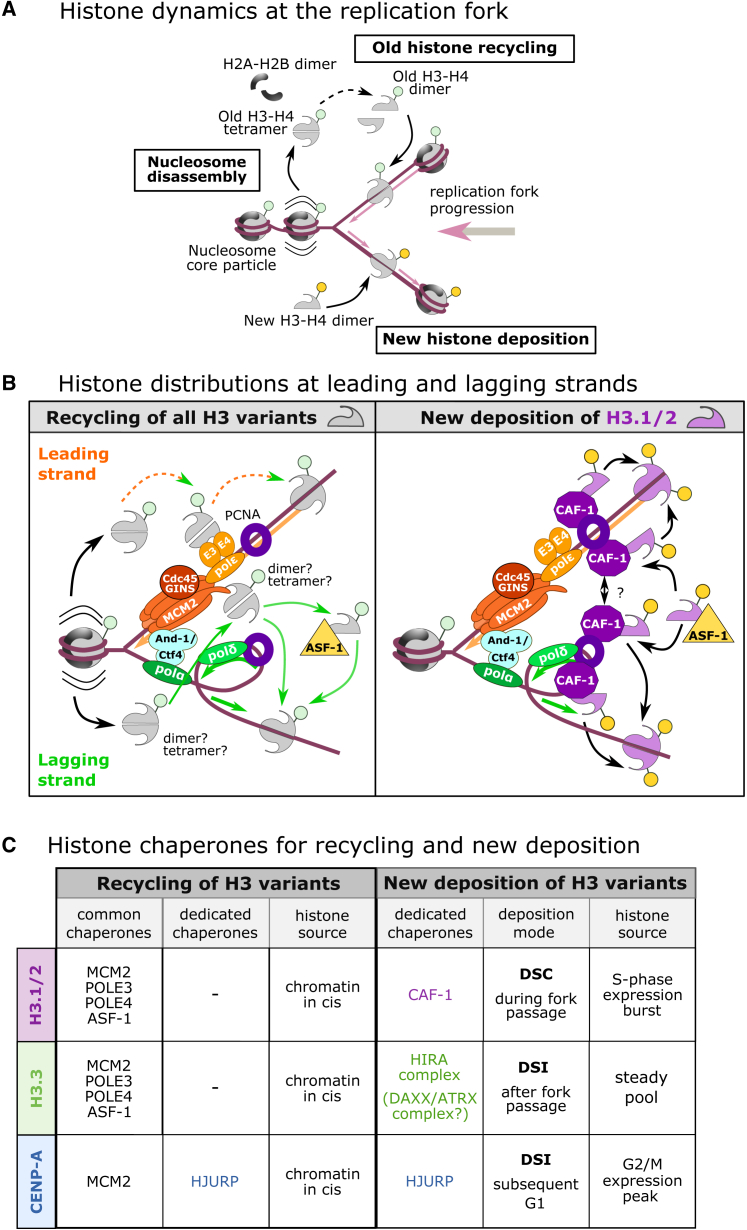
Histone dynamics during DNA replication (A) Scheme to highlight disassembly, recycling, and new deposition. (B) Recycling (left) of all H3 variants on the leading strand involves POLE3/4 with Pol epsilon and on the lagging strand MCM2 with Pol alpha and Pol gamma; ASF-1 can be important in both cases. New deposition (right) of H3.1/2 only is shown here since it is mediated by CAF-1 through its specific PCNA binding acting on both leading and lagging strands in a manner coupled to DNA synthesis; we hypothesize in this scheme how the dimerization of the largest subunit of CAF-1 can potentially contribute both to ensure the deposition of a tetramers and perhaps to connect leading and lagging strand deposition. (C) Summary of histone chaperones involved in recycling (left) and new deposition (right).

While transcription^66^ and DNA damage repair^67^ have been linked to local chromatin dynamics, replication is the most disruptive process genome-wide. The doubling of genetic information during S-phase requires replicating not only DNA but the entire chromatin. This uses both old and new histones in two main processes, i.e., histone recycling and *de novo* deposition (Figure 4A). Recycling of evicted histones onto newly synthesized DNA helps to preserve PTM patterns and chromatin states^68^ (Figure 4A). General machinery components including human and yeast MCM2,^69^^,^^70^ polymerase ε,^71^^,^^72^ and ASF-1^73^ (Figure 4C, left) have histone chaperone properties and form a platform to retain all variants. By contrast, newly synthesized histones (with their own sets of modifications) ensure the complement needed to maintain nucleosome density on doubling DNA (Figure 4A), and variant-dedicated chaperone complexes are solely responsible for deposition of newly synthesized H3 variants (Figure 4C, right). Marking with new histones may be a way to distinguish replicated from non-replicated regions, a possible means to avoid re-replication. While replication-coupled new deposition relies on replicative H3.1/2, recycling depends on any pre-existing variants at the given genomic location.

For replication to progress, the Cdc45-MCM(2-7)-GINS (MCM) helicase unwinds the DNA, making it accessible for DNA polymerases^74^ (Figure 4B). On the leading strand, DNA is synthesized in a continuous manner following the progressing fork, while lagging strand synthesis occurs in the opposite direction in short segments called Okazaki fragments.^74^ MCM2, as a subunit of the MCM helicase, may directly bind either the parental (H3-H4)_2_ tetramer or H3-H4 dimers^70^^,^^75^ in an H3-agnostic manner for recycling on the lagging strand^70^^,^^76^ (Figure 4B, left). On the leading strand, recycling is handled by subunits of polymerase ε (yeast Dpb3-Dpb4, human POLE3-POLE4)^72^^,^^77^ (Figure 4B, left). On the lagging strand, recycling depends on MCM2—either loading the entire tetramer back onto DNA via the MCM2-CTF4-Polα complex (an activity conserved from yeast to human)^78^ or loading H3-H4 dimers in concert with ASF-1^70^ (Figure 4B, left). ASF-1, the first chaperone linked to histone recycling during replication,^79^ can disrupt the histone tetramer^73^^,^^80^^,^^81^ for downstream dimer re-association (Figure 4B, left). Of note, mixed nucleosomes have only been observed with H3.3-H4 and not with H3.1-H4^82^—this may actually relate to the distinct deposition of H3.3 after the fork (see later).

Contrary to recycling, new deposition coupled to DNA synthesis so far has only been reported for the replicative histones H3.1/2. This deposition, occurring on both leading and lagging strands, is promoted by the interaction between CAF-1^41^ and proliferating cell nuclear antigen (PCNA)^83^ (Figure 4B, right). CAF-1 subunit p150 is phosphorylated^84^ and dimerizes^85^ to coordinate to both DNA strands, where it binds H3.1-H4 dimers.^21^^,^^86^^,^^87^ To form a complete tetramer, two CAF-1-H3.1-H4 complexes may bind a single trimeric PCNA to bring the two dimers together.^86^ Furthermore, CAF-1 may use distinct mechanisms on the leading and lagging strands.^88^ The paper clip (trombone) replication model proposes that looping of the lagging strand could coordinate not only replication between the strands^89^ (Figure 4B) but also histone deposition machinery and other mechanisms at the fork. For instance, a recent study indicates a crosstalk at the fork for histone modifications to re-establish post-replicative symmetry.^90^ Looping may also assist this replication-coupled epigenome maintenance.

Furthermore, DNA stability is constantly under threat as the double helix is unwound and duplicated, and histone dynamics and dosage play a role in protecting from and responding to replication stress. For instance, in yeast, histone levels have a direct relationship with DNA radiosensitivity.^3^^,^^91^ H3 in this system, where there are no other variants, play a role in recovering chromatin after replication stress through recombination-dependent replication (RDR). In this case, only CAF-1 and ASF-1 are required to mediate (H3-H4)_2_ tetramer deposition, while simultaneously conducting homologous repair; this effectively couples chromatin restoration and DNA repair.^92^ Species with H3.3 link this variant with genome stability as deficiency causes defects in fork progression in human cells^93^ and *C. elegans*,^94^ and H3.3 is required for fork restart.^95^ Similarly, CENP-A bolsters genome integrity as insufficient levels of this variant in S-phase impeded replication fork progression and increased R loops.^5^ Taken together, this shows that H3 variants are imperative for preventing and/or recovering from replication stress.

However, even when progressing faithfully, the replisome is disruptive. Even though most histones are recycled, one cannot ignore that DSC new deposition uses essentially replicative histones to fill in the doubling chromatin. This means the other variants are diluted following the passage of the replication fork, leaving the need to restore epigenomic states.

## Recovery of chromatin states

**Figure 5:**
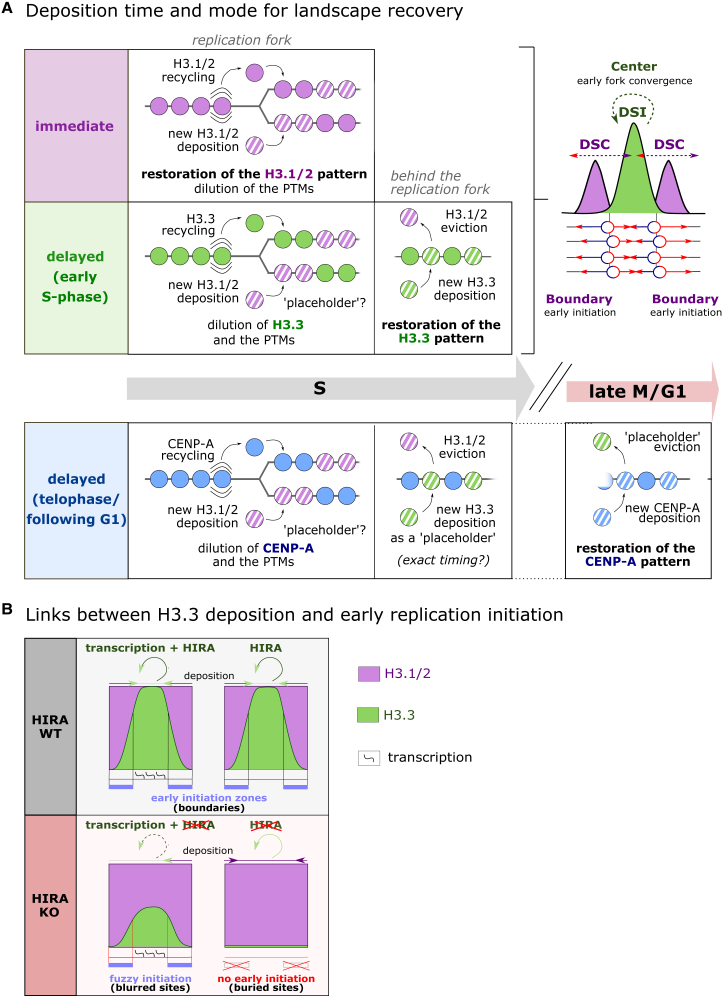
Recovery of a defined variant landscape after S-phase dilution We show simplified extreme scenarios with only one type of variant for the parental state. (A) Scenario summary of dilution and recovery dynamics for H3.1/2 (top), H3.3 (middle), and CENP-A (bottom). Right: scheme illustrating how the two distinct pathways, DSI deposition for H3.3 systematically at the same place combined with DSC deposition of H3.1, following the replication fork, create boundaries at early initiation zones of replication. (B) Scheme depicting how HIRA removal, and by extension removal of HIRA-dependent H3.3 deposition, affects not only deposition but also early replication zones. Two types of effects occur, and they distinguish two types of regions: blurred sites show blurred incorporation and blurred initiation zones at regions with active transcription, and buried sites correspond to regions without significant transcription where incorporation of H3.3 is abrogated, and the initiation zone disappears.

H3.1/2 acts as a “transient placeholder” or replacement for the other H3 variants during DNA synthesis, leading to a need to restore variant densities after fork progression. Following the progression of the replication fork, the recovery of each variant at local environments entails distinct processes. Here, we describe scenarios for three extreme situations. In the case of a track of nucleosome containing replicative H3.1/2, the local landscape restoration is immediate since both recycling and new deposition at the replication fork operate in a coordinated manner, and there is no local dilution of the variant (Figure 5A, top). Considering tracks containing the other H3 variants, even if recycled, these experience an initial local dilution as active deposition of newly synthesized replicative H3.1/2 is coupled to DNA synthesis (Figure 5A, mid/bottom). In the case of H3.3, the restoration pattern, while delayed, still occurs during S-phase. This is decoupled from DNA synthesis but still occurs in S-phase and enables repopulation with H3.3 at regions where H3.3 enrichment was detected before DNA replication.^96^ CENP-A restoration takes much longer to complete. Indeed, DNA replication dilutes CENP-A enrichment first with newly synthesized replicative H3.1/2 (Figure 5A, bottom) and later potentially with H3.3, as H3.3 may mark labile nucleosomes and function as a major “placeholder” for CENP-A.^37^ This state is maintained until late mitosis/G1, when new CENP-A deposition finally restores the centromere^24^ (Figure 5A, bottom).

Beyond restoration dynamics, an interesting question is whether histone deposition itself can in turn impact replication. To investigate this, novel SNAP-seq technology allowed for genome-wide mapping of new H3.1 and H3.3 deposition during early S-phase. This approach showed that new deposition of H3.1, linked to the replication fork, changed the landscape, while H3.3 deposition, returning to regions previously enriched with the non-replicative variant, depended on HIRA and was conservative.^96^ Interestingly, incorporation of these variants created H3.1/H3.3 boundaries corresponding to initiation zones of early-firing origins^96^ (Figure 5A, right). Remarkably, disruption of HIRA activity resulted in dysfunction of early replication zones with either (1) a loss of precision in both H3.3 deposition and replication initiation at regions with active transcription, termed “blurred”; or (2) a complete disappearance of H3.3 and early initiation at low or untranscribed regions, coined “buried”^96^ (Figure 5B). These phenotypes in early replication zones affecting initiation but not elongation reveal that H3 variant landscape is not a mere consequence of the deposition mechanisms linked to replication but may also have a regulatory role for the replication process itself. In considering the distinct functional roles for the variants, importance in humans is best highlighted by their phenotypes in disease.

## H3 variants in CIN and disease

Several studies have underlined the relations between H3 variants and CIN, a hallmark of disease and cancer. In H3.3^−/−^ ES cells,^4^ mitotic delays, and missegregated/lagging chromosomes are common phenotypes associated with disruption of H3.3 function. We have noted that H3.3S31 is a functional residue distinguishing this variant from the replicative H3; indeed, in *Xenopus* embryos, H3.3 depletion by morpholinos (MOs) results in gastrulation defects, and rescue experiments with H3.3S31A do not improve this phenotype when compared with wild type.^15^ However, while the mechanism driving this dysfunction is not characterized, the detection of apoptosis in H3.3 MO is reminiscent of the mouse H3.3 knockout (KO) partially rescued by p53 loss.^4^ This suggests possible connection with p53. Furthermore, there are several clues connecting H3.3, and S31 specifically with CIN. In particular, S31ph triggered a “proximity sensor” as it decorated misaligned chromosomes and led to p53 activation in primate cells.^97^ This modification is also upregulated in alternative lengthening of telmomeres positive (ALT+) cancers,^98^ where H3.3S31A expression increases DNA damage. Chromosome missegregation also occurs in human cells expressing the oncohistone mutation H3.3K27M.^99^ Notably, loss of CENP-A also leads to aneuploidy,^5^ consistent with its function at the centromere. Interestingly, CENP-A overexpression, as often observed in cancer^100^ (Table 1), also leads to mitotic defects.^101^ Not enough or too much proves deleterious. Furthermore, germline mutations in both human H3.3 genes, *H3F3A* and *H3F3B*, are linked with neurodegenerative disorders, without detection of cancer in these patients,^102^^,^^103^ including changes of key residues S31, G34, and G90 (Table 1). A case of a newly identified mutation in H3.3, L62R (Table 1), may act as a driver in microcephaly,^104^ another disease characterized by chromatin instability.

**Table 1:** Summary of identified H3 variant perturbations in disease

Disease	Median age at diagnosis (pediatric/adult)	H3 variant aa substitutions (somatic, germ line)/alterations (% frequency)	References
Microcephaly	<1 year	changes throughout coding sequence (e.g., H3.3S31F, H3.3G34V, H3.3L62R, H3.3G90R)	Bryant et al.,^102^ Okur er al.,^103^ and Maver et al.^104^
Diffuse intrinsic pontine glioma (DIPG)	12 years	H3.1K27M (31%), H3.3K27M (93%), H3.3K27I (<1%)	Schwartzentruber et al.,^105^ Mohammad and Helin,^106^ Nacev et al.,^107^ Verschoor et al.,^108^ Crowell et al.,^109^ and Erker et al.^110^
Diffuse hemispheric glioma (DHG)	16 years	H3.3G34R/V (15%)	
Giant cell tumors of bone (GCTB)	35 years	H3.3G34W/L (92%)	
Chondroblastoma	21 years	H3.3K36M (95%)	
Cancer (breast, liver, ovarian, colon, bone, gastric, prostate)	adult	CENP-A overexpression (44%)	Renaud-Pageot et al.,^100^ Sharma et al.,^111^ and Jeffery et al.^112^

These include microcephaly,^102^^,^^103^^,^^104^ oncohistone cancers: diffuse intrinsic pontine glioma (DIPG), diffuse hemispheric glioma (DHG), giant cell tumors of bone (GCTB), chondroblastoma,^105^^,^^106^^,^^107^^,^^108^^,^^109^^,^^110^ and cancers with CENP-A overexpression.^100^^,^^111^^,^^112^

“Oncohistone” mutations—somatic, heterozygous gain-of-function mutations in H3 encoding genes—have gained attention for their driving potential in cancer^105^^,^^106^^,^^107^^,^^113^ (Table 1), particularly K27M.^114^^,^^115^ Oncohistone KtoM substitutions drastically ablate associated methylations^116^^,^^117^^,^^118^^,^^119^^,^^120^ with clear detrimental effects on the epigenome. This draws an obvious link with gene expression, which has been explored in several systems. For instance, in mouse embryonic stem cells (mESCs), H3.3K9M or H3.3K36M expression was sufficient to disrupt hematopoietic differentiation potentially due to the altered gene expression.^121^ In mouse germline cells, isogenic direct knockin G34R/V/W mutants also caused large transcription profile changes. This is resulting from global loss of H3K36 methylation, leading to impinged DNMT3A recruitment and causing neuronal degeneration, neuroinflammation, and developmental delay.^122^ However, in a recent study using mESCs, extensive CRISPR efforts changing each H3.1/3.2/3.3 gene to K27R found this residue was ultimately dispensable for transcriptional binding and activation.^123^ This suggests that detrimental effects of oncohistones may reach beyond transcription phenotypes. This is in agreement with what was reported in *Drosophila*, where after tracking H3.1K327M and H3.3K27M, effects were restricted to cycling cells,^124^ indicating an effect on replication-dependent chromatin states. These data further exemplify the critical role of H3.3 in humans and disease.

Altered chromatin landscapes may also result from promiscuous interactions between H3 variants and chaperones arising from unbalanced dosage situations. One striking example results from CENP-A overexpression, observed in many solid and aggressive cancers.^100^ Under normal conditions, CENP-A expression and HJURP expression are tightly co-regulated and their interaction ensures their stability.^125^ However, when overexpressed, CENP-A can associate with DAXX and/or HIRA, which in turn promotes ectopic incorporation into chromosomal arms.^33^^,^^126^^,^^127^ On the one hand, CENP-A enrichment outside of the centromere may weaken centromere function by directing components of kinetochore machinery away from the centromere, leading to mitotic defects and CIN.^101^^,^^128^ On the other hand, incorporation of CENP-A at H3.3 sites can also potentiate genome function, an aspect that is still under investigation. Lastly, regulation of H3.1/2 deposition by CAF-1 activity is suppressed in breast cancer during metastasis, accompanied by increased HIRA-mediated H3.3 deposition, leading to epithelial-mesenchymal transition.^129^ Thus, it is important to consider an overall equilibrium between all three variants and their chaperones to appreciate their impacts on cell function.

## Conclusions and future perspectives

Here, we summarized our current knowledge concerning the functional differences between the H3 variants in terms of expression, sequence, new deposition, and recycling. We described their genomic enrichment at distinct loci and how dedicated chaperone complexes contribute to these distributions. The comparison of UBN1 and DAXX orthologs across different species reveals a co-evolution in DAXX at critical residues for H3.3 specific recognition in species where H3.3 is essential. We provided the current view for the dynamics of the variants at the replication fork, as well as how the recovery of chromatin states follows this disruptive event to coordinate both recycling and new deposition. We then considered how mutations and dysregulation affect histone variants in human disease. We thus stressed how similar mutations may have different outcomes in terms of diseases, according to the context, i.e., where and when and germ line versus somatic mutations. We stressed the importance of proper chromatin assembly and provided contexts in which each variant contributes to genome stability.

This review has laid a foundation for understanding H3 variant and chaperone dynamics in a basal setting, but ongoing work is needed to consistently push for exploring how these may change and adapt under stressful conditions. For instance, histone dosage changes may operate to compensate and restore homeostasis, yet imbalance may be detrimental to the cell. Even more, imparity between chaperones may achieve similar effects to variant dosages or may regulate variants in a manner yet to be determined. Furthermore, this review has not considered chaperones upstream of those depositing histones into chromatin, nor those regulating the expression of histones, which themselves are interesting parameters to consider with respect to how their activity and/or disparity impacts the global chromatin network. Understanding the dynamics herein will help to clarify the boundaries between compensatory and pathogenic, thereby defining new markers of disease and possibly leading to new targets for therapy. With the clear link between genome integrity and disease, here we have provided another link in the chain in outlining the importance of the H3 family to chromatin integrity.

## References

[1] Yadav T., Quivy J.P., Almouzni G. (2018). Chromatin plasticity: a versatile landscape that underlies cell fate and identity. Science.

[2] Gunjan A., Verreault A. (2003). A Rad53 kinase-dependent surveillance mechanism that regulates histone protein levels in S. cerevisiae. Cell.

[3] Liang D., Burkhart S.L., Singh R.K., Kabbaj M.H., Gunjan A. (2012). Histone dosage regulates DNA damage sensitivity in a checkpoint-independent manner by the homologous recombination pathway. Nucleic Acids Res..

[4] Jang C.W., Shibata Y., Starmer J., Yee D., Magnuson T. (2015). Histone H3.3 maintains genome integrity during mammalian development. Genes Dev..

[5] Giunta S., Hervé S., White R.R., Wilhelm T., Dumont M., Scelfo A., Gamba R., Wong C.K., Rancati G., Smogorzewska A. (2021). CENP-A chromatin prevents replication stress at centromeres to avoid structural aneuploidy. Proc. Natl. Acad. Sci. USA.

[6] Szenker E., Ray-Gallet D., Almouzni G. (2011). The double face of the histone variant H3.3. Cell Res..

[7] Molaro A., Drinnenberg I.A., Orsi G.A., Almouzni G. (2018). Histone Variants Methods in Molecular Biology.

[8] Franklin S.G., Zweidler A. (1977). Non-allelic variants of histones 2a, 2b and 3 in mammals. Nature.

[9] Wu R.S., Bonner W.M. (1981). Separation of basal histone synthesis from S-phase histone synthesis in dividing cells. Cell.

[10] Shelby R.D., Vafa O., Sullivan K.F. (1997). Assembly of CENP-A into centromeric chromatin requires a cooperative array of nucleosomal DNA contact sites. J. Cell Biol..

[11] Shelby R.D., Monier K., Sullivan K.F. (2000). Chromatin assembly at kinetochores is uncoupled from DNA replication. J. Cell Biol..

[12] Maze I., Noh K.M., Soshnev A.A., Allis C.D. (2014). Every amino acid matters: essential contributions of histone variants to mammalian development and disease. Nat. Rev. Genet..

[13] Martire S., Gogate A.A., Whitmill A., Tafessu A., Nguyen J., Teng Y.C., Tastemel M., Banaszynski L.A. (2019). Phosphorylation of histone H3.3 at serine 31 promotes p300 activity and enhancer acetylation. Nat. Genet..

[14] Armache A., Yang S., Martínez de Paz A., Robbins L.E., Durmaz C., Cheong J.Q., Ravishankar A., Daman A.W., Ahimovic D.J., Klevorn T. (2020). Histone H3.3 phosphorylation amplifies stimulation-induced transcription. Nature.

[15] Sitbon D., Boyarchuk E., Dingli F., Loew D., Almouzni G. (2020). Histone variant H3.3 residue S31 is essential for Xenopus gastrulation regardless of the deposition pathway. Nat. Commun..

[16] Talbert P.B., Bryson T.D., Henikoff S. (2004). Adaptive evolution of centromere proteins in plants and animals. J. Biol..

[17] Dunleavy E.M., Roche D., Tagami H., Lacoste N., Ray-Gallet D., Nakamura Y., Daigo Y., Nakatani Y., Almouzni-Pettinotti G. (2009). HJURP is a cell-cycle-dependent maintenance and deposition factor of CENP-A at centromeres. Cell.

[18] Foltz D.R., Jansen L.E.T., Bailey A.O., Yates J.R., Bassett E.A., Wood S., Black B.E., Cleveland D.W. (2009). Centromere-specific assembly of CENP-A nucleosomes is mediated by HJURP. Cell.

[19] Black B.E., Foltz D.R., Chakravarthy S., Luger K., Woods V.L., Cleveland D.W. (2004). Structural determinants for generating centromeric chromatin. Nature.

[20] Latreille D., Bluy L., Benkirane M., Kiernan R.E. (2014). Identification of histone 3 variant 2 interacting factors. Nucleic Acids Res..

[21] Tagami H., Ray-Gallet D., Almouzni G., Nakatani Y. (2004). Histone H3.1 and H3.3 complexes mediate nucleosome assembly pathways dependent or independent of DNA synthesis. Cell.

[22] Ahmad K., Henikoff S. (2002). The histone variant H3.3 marks active chromatin by replication-independent nucleosome assembly. Mol. Cell.

[23] Torné J., Orsi G.A., Ray-Gallet D., Almouzni G., Orsi G.A., Almouzni G. (2018). Histone Variants: Methods and Protocols Methods in Molecular Biology.

[24] Jansen L.E.T., Black B.E., Foltz D.R., Cleveland D.W. (2007). Propagation of centromeric chromatin requires exit from mitosis. J. Cell Biol..

[25] Stirpe A., Heun P. (2023). The ins and outs of CENP-A: chromatin dynamics of the centromere-specific histone. Semin. Cell Dev. Biol..

[26] Clément C., Orsi G.A., Gatto A., Boyarchuk E., Forest A., Hajj B., Miné-Hattab J., Garnier M., Gurard-Levin Z.A., Quivy J.P. (2018). High-resolution visualization of H3 variants during replication reveals their controlled recycling. Nat. Commun..

[27] Liang K., Woodfin A.R., Slaughter B.D., Unruh J.R., Box A.C., Rickels R.A., Gao X., Haug J.S., Jaspersen S.L., Shilatifard A. (2015). Mitotic transcriptional activation: clearance of actively engaged Pol II via transcriptional elongation control in mitosis. Mol. Cell.

[28] Dellino G.I., Cittaro D., Piccioni R., Luzi L., Banfi S., Segalla S., Cesaroni M., Mendoza-Maldonado R., Giacca M., Pelicci P.G. (2013). Genome-wide mapping of human DNA-replication origins: levels of transcription at ORC1 sites regulate origin selection and replication timing. Genome Res..

[29] Lund E., Oldenburg A.R., Delbarre E., Freberg C.T., Duband-Goulet I., Eskeland R., Buendia B., Collas P. (2013). Lamin A/C-promoter interactions specify chromatin state–dependent transcription outcomes. Genome Res..

[30] Elsässer S.J., Noh K.M., Diaz N., Allis C.D., Banaszynski L.A. (2015). Histone H3.3 is required for endogenous retroviral element silencing in embryonic stem cells. Nature.

[31] Wong L.H., Ren H., Williams E., McGhie J., Ahn S., Sim M., Tam A., Earle E., Anderson M.A., Mann J. (2009). Histone H3.3 incorporation provides a unique and functionally essential telomeric chromatin in embryonic stem cells. Genome Res..

[32] Probst A.V., Dunleavy E., Almouzni G. (2009). Epigenetic inheritance during the cell cycle. Nat. Rev. Mol. Cell Biol..

[33] Lacoste N., Woolfe A., Tachiwana H., Garea A.V., Barth T., Cantaloube S., Kurumizaka H., Imhof A., Almouzni G. (2014). Mislocalization of the centromeric histone variant CenH3/CENP-A in human cells depends on the chaperone DAXX. Mol. Cell.

[34] Arunkumar G., Melters D.P. (2020). Centromeric transcription: a conserved Swiss-army knife. Genes.

[35] Altemose N., Logsdon G.A., Bzikadze A.V., Sidhwani P., Langley S.A., Caldas G.V., Hoyt S.J., Uralsky L., Ryabov F.D., Shew C.J. (2022). Complete genomic and epigenetic maps of human centromeres. Science.

[36] Gershman A., Sauria M.E.G., Guitart X., Vollger M.R., Hook P.W., Hoyt S.J., Jain M., Shumate A., Razaghi R., Koren S. (2022). Epigenetic patterns in a complete human genome. Science.

[37] Dunleavy E.M., Almouzni G., Karpen G.H. (2011). H3.3 is deposited at centromeres in S phase as a placeholder for newly assembled CENP-A in G₁ phase. Nucleus.

[38] Ricketts M.D., Frederick B., Hoff H., Tang Y., Schultz D.C., Singh Rai T., Grazia Vizioli M., Adams P.D., Marmorstein R. (2015). Ubinuclein-1 confers histone H3.3-specific-binding by the HIRA histone chaperone complex. Nat. Commun..

[39] Liu C.P., Xiong C., Wang M., Yu Z., Yang N., Chen P., Zhang Z., Li G., Xu R.M. (2012). Structure of the variant histone H3.3-H4 heterodimer in complex with its chaperone DAXX. Nat. Struct. Mol. Biol..

[40] Hu H., Liu Y., Wang M., Fang J., Huang H., Yang N., Li Y., Wang J., Yao X., Shi Y. (2011). Structure of a CENP-A–histone H4 heterodimer in complex with chaperone HJURP. Genes Dev..

[41] Smith S., Stillman B. (1989). Purification and characterization of CAF-I, a human cell factor required for chromatin assembly during DNA replication in vitro. Cell.

[42] Ray-Gallet D., Almouzni G., Fang D., Han J. (2021). Histone Mutations and Cancer Advances in Experimental Medicine and Biology.

[43] Taddei A., Roche D., Sibarita J.B., Turner B.M., Almouzni G. (1999). Duplication and maintenance of heterochromatin domains. J. Cell Biol..

[44] Adam S., Dabin J., Chevallier O., Leroy O., Baldeyron C., Corpet A., Lomonte P., Renaud O., Almouzni G., Polo S.E. (2016). Real-time tracking of parental histones reveals their contribution to chromatin integrity following DNA damage. Mol. Cell.

[45] Ray-Gallet D., Quivy J.P., Scamps C., Martini E.M.-D., Lipinski M., Almouzni G. (2002). HIRA is critical for a nucleosome assembly pathway independent of DNA synthesis. Mol. Cell.

[46] Drané P., Ouararhni K., Depaux A., Shuaib M., Hamiche A. (2010). The death-associated protein DAXX is a novel histone chaperone involved in the replication-independent deposition of H3.3.. Genes Dev..

[47] Goldberg A.D., Banaszynski L.A., Noh K.M., Lewis P.W., Elsaesser S.J., Stadler S., Dewell S., Law M., Guo X., Li X. (2010). Distinct factors control histone variant H3.3 localization at specific genomic regions. Cell.

[48] Elsässer S.J., Huang H., Lewis P.W., Chin J.W., Allis C.D., Patel D.J. (2012). DAXX envelops a histone H3.3-H4 dimer for H3.3-specific recognition. Nature.

[49] Elsaesser S.J., Allis C.D. (2010). HIRA and Daxx constitute two independent histone H3.3-containing predeposition complexes. Cold Spring Harb. Symp. Quant. Biol..

[50] Banumathy G., Somaiah N., Zhang R., Tang Y., Hoffmann J., Andrake M., Ceulemans H., Schultz D., Marmorstein R., Adams P.D. (2009). Human UBN1 is an ortholog of yeast Hpc2p and has an essential role in the HIRA/ASF1a chromatin-remodeling pathway in senescent cells. Mol. Cell. Biol..

[51] Rai T.S., Puri A., McBryan T., Hoffman J., Tang Y., Pchelintsev N.A., van Tuyn J., Marmorstein R., Schultz D.C., Adams P.D. (2011). Human CABIN1 is a functional member of the human HIRA/UBN1/ASF1a histone H3.3 chaperone complex. Mol. Cell. Biol..

[52] Ray-Gallet D., Ricketts M.D., Sato Y., Gupta K., Boyarchuk E., Senda T., Marmorstein R., Almouzni G. (2018). Functional activity of the H3.3 histone chaperone complex HIRA requires trimerization of the HIRA subunit. Nat. Commun..

[53] Ricketts M.D., Dasgupta N., Fan J., Han J., Gerace M., Tang Y., Black B.E., Adams P.D., Marmorstein R. (2019). The HIRA histone chaperone complex subunit UBN1 harbors H3/H4- and DNA-binding activity. J. Biol. Chem..

[54] Torné J., Ray-Gallet D., Boyarchuk E., Garnier M., Le Baccon P., Coulon A., Orsi G.A., Almouzni G. (2020). Two HIRA-dependent pathways mediate H3.3 de novo deposition and recycling during transcription. Nat. Struct. Mol. Biol..

[55] Lewis P.W., Elsaesser S.J., Noh K.M., Stadler S.C., Allis C.D. (2010). Daxx is an H3.3-specific histone chaperone and cooperates with ATRX in replication-independent chromatin assembly at telomeres. Proc. Natl. Acad. Sci. USA.

[56] Voon H.P.J., Hughes J.R., Rode C., De La Rosa-Velázquez I.A., Jenuwein T., Feil R., Higgs D.R., Gibbons R.J. (2015). ATRX plays a key role in maintaining silencing at interstitial heterochromatic loci and imprinted genes. Cell Rep..

[57] Wasylishen A.R., Sun C., Moyer S.M., Qi Y., Chau G.P., Aryal N.K., McAllister F., Kim M.P., Barton M.C., Estrella J.S. (2020). Daxx maintains endogenous retroviral silencing and restricts cellular plasticity in vivo. Sci. Adv..

[58] Carraro M., Hendriks I.A., Hammond C.M., Solis-Mezarino V., Völker-Albert M., Elsborg J.D., Weisser M.B., Spanos C., Montoya G., Rappsilber J. (2023). DAXX adds a de novo H3.3K9me3 deposition pathway to the histone chaperone network. Mol. Cell.

[59] Udugama M., Chang F.T.M., Chan F.L., Tang M.C., Pickett H.A., McGhie J.D.R., Mayne L., Collas P., Mann J.R., Wong L.H. (2015). Histone variant H3.3 provides the heterochromatic H3 lysine 9 tri-methylation mark at telomeres. Nucleic Acids Res..

[60] Gerber J.P., Russ J., Chandrasekar V., Offermann N., Lee H.M., Spear S., Guzzi N., Maida S., Pattabiraman S., Zhang R. (2021). Aberrant chromatin landscape following loss of the H3.3 chaperone Daxx in haematopoietic precursors leads to Pu.1-mediated neutrophilia and inflammation. Nat. Cell Biol..

[61] Tian Q., Wang X.F., Xie S.M., Yin Y., Zhou L.Q. (2020). H3.3 impedes zygotic transcriptional program activated by Dux. Biochem. Biophys. Res. Commun..

[62] Udugama M., Vinod B., Chan F.L., Hii L., Garvie A., Collas P., Kalitsis P., Steer D., Das P.P., Tripathi P. (2022). Histone H3.3 phosphorylation promotes heterochromatin formation by inhibiting H3K9/K36 histone demethylase. Nucleic Acids Res..

[63] Zasadzińska E., Huang J., Bailey A.O., Guo L.Y., Lee N.S., Srivastava S., Wong K.A., French B.T., Black B.E., Foltz D.R. (2018). Inheritance of CENP-A nucleosomes during DNA replication requires HJURP. Dev. Cell.

[64] Smith M.M., Stirling V.B. (1988). Histone H3 and H4 gene deletions in Saccharomyces cerevisiae. J. Cell Biol..

[65] Huang S., Zhou H., Katzmann D., Hochstrasser M., Atanasova E., Zhang Z. (2005). Rtt106p is a histone chaperone involved in heterochromatin-mediated silencing. Proc. Natl. Acad. Sci. USA.

[66] Delaney K., Almouzni G. (2023). Transcription-coupled H3.3 recycling: a link with chromatin states. Semin. Cell Dev. Biol..

[67] Polo S.E., Almouzni G. (2015). Chromatin dynamics after DNA damage: the legacy of the access–repair–restore model. DNA Repair.

[68] Stewart-Morgan K.R., Petryk N., Groth A. (2020). Chromatin replication and epigenetic cell memory. Nat. Cell Biol..

[69] Foltman M., Evrin C., De Piccoli G., Jones R.C., Edmondson R.D., Katou Y., Nakato R., Shirahige K., Labib K. (2013). Eukaryotic replisome components cooperate to process histones during chromosome replication. Cell Rep..

[70] Huang H., Strømme C.B., Saredi G., Hödl M., Strandsby A., González-Aguilera C., Chen S., Groth A., Patel D.J. (2015). A unique binding mode enables MCM2 to chaperone histones H3-H4 at replication forks. Nat. Struct. Mol. Biol..

[71] Bellelli R., Belan O., Pye V.E., Clement C., Maslen S.L., Skehel J.M., Cherepanov P., Almouzni G., Boulton S.J. (2018). POLE3-POLE4 is a histone H3-H4 chaperone that maintains chromatin integrity during DNA replication. Mol. Cell.

[72] Yu C., Gan H., Serra-Cardona A., Zhang L., Gan S., Sharma S., Johansson E., Chabes A., Xu R.M., Zhang Z. (2018). A mechanism for preventing asymmetric histone segregation onto replicating DNA strands. Science.

[73] English C.M., Adkins M.W., Carson J.J., Churchill M.E.A., Tyler J.K. (2006). Structural basis for the histone chaperone activity of Asf1. Cell.

[74] Riera A., Barbon M., Noguchi Y., Reuter L.M., Schneider S., Speck C. (2017). From structure to mechanism-understanding initiation of DNA replication. Genes Dev..

[75] Richet N., Liu D., Legrand P., Velours C., Corpet A., Gaubert A., Bakail M., Moal-Raisin G., Guerois R., Compper C. (2015). Structural insight into how the human helicase subunit MCM2 may act as a histone chaperone together with ASF1 at the replication fork. Nucleic Acids Res..

[76] Xu X., Duan S., Hua X., Li Z., He R., Zhang Z. (2022). Stable inheritance of H3.3-containing nucleosomes during mitotic cell divisions. Nat. Commun..

[77] Li Z., Hua X., Serra-Cardona A., Xu X., Gan S., Zhou H., Yang W.S., Chen C.L., Xu R.M., Zhang Z. (2020). DNA polymerase α interacts with H3-H4 and facilitates the transfer of parental histones to lagging strands. Sci. Adv..

[78] Gan H., Serra-Cardona A., Hua X., Zhou H., Labib K., Yu C., Zhang Z. (2018). The Mcm2-Ctf4-Polα axis facilitates parental histone H3-H4 transfer to lagging strands. Mol. Cell.

[79] Groth A., Corpet A., Cook A.J.L., Roche D., Bartek J., Lukas J., Almouzni G. (2007). Regulation of replication fork progression through histone supply and demand. Science.

[80] Mousson F., Lautrette A., Thuret J.Y., Agez M., Courbeyrette R., Amigues B., Becker E., Neumann J.M., Guerois R., Mann C. (2005). Structural basis for the interaction of Asf1 with histone H3 and its functional implications. Proc. Natl. Acad. Sci. USA.

[81] Natsume R., Eitoku M., Akai Y., Sano N., Horikoshi M., Senda T. (2007). Structure and function of the histone chaperone CIA/ASF1 complexed with histones H3 and H4. Nature.

[82] Xu M., Long C., Chen X., Huang C., Chen S., Zhu B. (2010). Partitioning of histone H3-H4 tetramers during DNA replication-dependent chromatin assembly. Science.

[83] Shibahara K., Stillman B. (1999). Replication-dependent marking of DNA by PCNA facilitates CAF-1-coupled inheritance of chromatin. Cell.

[84] Gérard A., Koundrioukoff S., Ramillon V., Sergère J.C., Mailand N., Quivy J.P., Almouzni G. (2006). The replication kinase Cdc7-Dbf4 promotes the interaction of the p150 subunit of chromatin assembly factor 1 with proliferating cell nuclear antigen. EMBO Rep..

[85] Quivy J.P., Grandi P., Almouzni G. (2001). Dimerization of the largest subunit of chromatin assembly factor 1: importance in vitro and during Xenopus early development. EMBO J..

[86] Mattiroli F., Gu Y., Yadav T., Balsbaugh J.L., Harris M.R., Findlay E.S., Liu Y., Radebaugh C.A., Stargell L.A., Ahn N.G. (2017). DNA-mediated association of two histone-bound complexes of yeast chromatin assembly Factor-1 (CAF-1) drives tetrasome assembly in the wake of DNA replication. eLife.

[87] Nakano S., Stillman B., Horvitz H.R. (2011). Replication-coupled chromatin assembly generates a neuronal bilateral asymmetry in C. elegans. Cell.

[88] Rouillon C., Eckhardt B.V., Kollenstart L., Gruss F., Verkennis A.E.E., Rondeel I., Krijger P.H.L., Ricci G., Biran A., van Laar T. (2023). CAF-1 deposits newly synthesized histones during DNA replication using distinct mechanisms on the leading and lagging strands. Nucleic Acids Res..

[89] Alberts B.M., Barry J., Bedinger P., Formosa T., Jongeneel C.V., Kreuzer K.N. (1983). Studies on DNA replication in the bacteriophage T4 in vitro system. Cold Spring Harb. Symp. Quant. Biol..

[90] Flury V., Reverón-Gómez N., Alcaraz N., Stewart-Morgan K.R., Wenger A., Klose R.J., Groth A. (2023). Recycling of modified H2A-H2B provides short-term memory of chromatin states. Cell.

[91] Singh R.K., Kabbaj M.H., Paik J., Gunjan A. (2009). Histone levels are regulated by phosphorylation and ubiquitylation-dependent proteolysis. Nat. Cell Biol..

[92] Hardy J., Dai D., Ait Saada A., Teixeira-Silva A., Dupoiron L., Mojallali F., Fréon K., Ochsenbein F., Hartmann B., Lambert S. (2019). Histone deposition promotes recombination-dependent replication at arrested forks. PLoS Genet..

[93] Frey A., Listovsky T., Guilbaud G., Sarkies P., Sale J.E. (2014). Histone H3.3 is required to maintain replication fork progression after UV damage. Curr. Biol..

[94] Strobino M., Wenda J.M., Padayachy L., Steiner F.A. (2020). Loss of histone H3.3 results in DNA replication defects and altered origin dynamics in C. elegans. Genome Res..

[95] Raghunandan M., Yeo J.E., Walter R., Saito K., Harvey A.J., Ittershagen S., Lee E.A., Yang J., Hoatlin M.E., Bielinsky A.K. (2020). Functional cross talk between the Fanconi anemia and ATRX/DAXX histone chaperone pathways promotes replication fork recovery. Hum. Mol. Genet..

[96] Gatto A., Forest A., Quivy J.P., Almouzni G. (2022). HIRA-dependent boundaries between H3 variants shape early replication in mammals. Mol. Cell.

[97] Hinchcliffe E.H., Day C.A., Karanjeet K.B., Fadness S., Langfald A., Vaughan K.T., Dong Z. (2016). Chromosome missegregation during anaphase triggers p53 cell cycle arrest through histone H3.3 Ser31 phosphorylation. Nat. Cell Biol..

[98] Chang F.T.M., Chan F.L., R McGhie J.D., Udugama M., Mayne L., Collas P., Mann J.R., Wong L.H. (2015). CHK1-driven histone H3.3 serine 31 phosphorylation is important for chromatin maintenance and cell survival in human ALT cancer cells. Nucleic Acids Res..

[99] Bočkaj I., Martini T.E.I., de Camargo Magalhães E.S., Bakker P.L., Meeuwsen-de Boer T.G.J., Armandari I., Meuleman S.L., Mondria M.T., Stok C., Kok Y.P. (2021). The H3.3K27M oncohistone affects replication stress outcome and provokes genomic instability in pediatric glioma. PLoS Genet..

[100] Renaud-Pageot C., Quivy J.P., Lochhead M., Almouzni G. (2022). CENP-A regulation and cancer. Front. Cell Dev. Biol..

[101] Shrestha R.L., Rossi A., Wangsa D., Hogan A.K., Zaldana K.S., Suva E., Chung Y.J., Sanders C.L., Difilippantonio S., Karpova T.S. (2021). CENP-A overexpression promotes aneuploidy with karyotypic heterogeneity. J. Cell Biol..

[102] Bryant L., Li D., Cox S.G., Marchione D., Joiner E.F., Wilson K., Janssen K., Lee P., March M.E., Nair D. (2020). Histone H3.3 beyond cancer: germline mutations in Histone 3 Family 3A and 3B cause a previously unidentified neurodegenerative disorder in 46 patients. Sci. Adv..

[103] Okur V., Chen Z., Vossaert L., Peacock S., Rosenfeld J., Zhao L., Du H., Calamaro E., Gerard A., Zhao S. (2021). De novo variants in H3-3A and H3-3B are associated with neurodevelopmental delay, dysmorphic features, and structural brain abnormalities. npj Genom. Med..

[104] Maver A., Čuturilo G., Ruml S.J., Peterlin B. (2019). Clinical next generation sequencing reveals an H3F3A gene as a new potential gene candidate for microcephaly associated with severe developmental delay, intellectual disability and growth retardation. Balkan J. Med. Genet..

[105] Schwartzentruber J., Korshunov A., Liu X.Y., Jones D.T.W., Pfaff E., Jacob K., Sturm D., Fontebasso A.M., Quang D.A., Tönjes M. (2012). Driver mutations in histone H3.3 and chromatin remodelling genes in paediatric glioblastoma. Nature.

[106] Mohammad F., Helin K. (2017). Oncohistones: drivers of pediatric cancers. Genes Dev..

[107] Nacev B.A., Feng L., Bagert J.D., Lemiesz A.E., Gao J., Soshnev A.A., Kundra R., Schultz N., Muir T.W., Allis C.D. (2019). The expanding landscape of ‘oncohistone’ mutations in human cancers. Nature.

[108] Verschoor A.J., Bovée J.V.M.G., Mastboom M.J.L., Sander Dijkstra P.D., Van De Sande M.A.J., Gelderblom H. (2018). Incidence and demographics of giant cell tumor of bone in the Netherlands: first nationwide Pathology Registry Study. Acta Orthop..

[109] Crowell C., Mata-Mbemba D., Bennett J., Matheson K., Mackley M., Perreault S., Erker C. (2022). Systematic review of diffuse hemispheric glioma, H3 G34-mutant: outcomes and associated clinical factors. Neurooncol. Adv..

[110] Erker C., Lane A., Chaney B., Leary S., Minturn J.E., Bartels U., Packer R.J., Dorris K., Gottardo N.G., Warren K.E. (2022). Characteristics of patients ≥10 years of age with diffuse intrinsic pontine glioma: a report from the International DIPG/DMG Registry. Neuro. Oncol.

[111] Sharma A.B., Dimitrov S., Hamiche A., Van Dyck E. (2019). Centromeric and ectopic assembly of CENP-A chromatin in health and cancer: old marks and new tracks. Nucleic Acids Res..

[112] Jeffery D., Lochhead M., Almouzni G., Kloc M., Kubiak J.Z. (2022). Results and Problems in Cell Differentiation.

[113] Wu G., Broniscer A., McEachron T.A., Lu C., Paugh B.S., Becksfort J., Qu C., Ding L., Huether R., Parker M. (2012). Somatic histone H3 alterations in pediatric diffuse intrinsic pontine gliomas and non-brainstem glioblastomas. Nat. Genet..

[114] Khuong-Quang D.A., Buczkowicz P., Rakopoulos P., Liu X.Y., Fontebasso A.M., Bouffet E., Bartels U., Albrecht S., Schwartzentruber J., Letourneau L. (2012). K27M mutation in histone H3.3 defines clinically and biologically distinct subgroups of pediatric diffuse intrinsic pontine gliomas. Acta Neuropathol..

[115] Larson J.D., Kasper L.H., Paugh B.S., Jin H., Wu G., Kwon C.H., Fan Y., Shaw T.I., Silveira A.B., Qu C. (2019). Histone H3.3 K27M accelerates spontaneous brainstem glioma and drives restricted changes in bivalent gene expression. Cancer Cell.

[116] Bender S., Tang Y., Lindroth A.M., Hovestadt V., Jones D.T.W., Kool M., Zapatka M., Northcott P.A., Sturm D., Wang W. (2013). Reduced H3K27me3 and DNA hypomethylation are major drivers of gene expression in K27M mutant pediatric high-grade gliomas. Cancer Cell.

[117] Chan K.M., Fang D., Gan H., Hashizume R., Yu C., Schroeder M., Gupta N., Mueller S., James C.D., Jenkins R. (2013). The histone H3.3K27M mutation in pediatric glioma reprograms H3K27 methylation and gene expression. Genes Dev..

[118] Lewis P.W., Müller M.M., Koletsky M.S., Cordero F., Lin S., Banaszynski L.A., Garcia B.A., Muir T.W., Becher O.J., Allis C.D. (2013). Inhibition of PRC2 activity by a gain-of-function H3 mutation found in pediatric glioblastoma. Science.

[119] Fang D., Gan H., Lee J.H., Han J., Wang Z., Riester S.M., Jin L., Chen J., Zhou H., Wang J. (2016). The histone H3.3K36M mutation reprograms the epigenome of chondroblastomas. Science.

[120] Lu C., Jain S.U., Hoelper D., Bechet D., Molden R.C., Ran L., Murphy D., Venneti S., Hameed M., Pawel B.R. (2016). Histone H3K36 mutations promote sarcomagenesis through altered histone methylation landscape. Science.

[121] Brumbaugh J., Kim I.S., Ji F., Huebner A.J., Di Stefano B., Schwarz B.A., Charlton J., Coffey A., Choi J., Walsh R.M. (2019). Inducible histone K-to-M mutations are dynamic tools to probe the physiological role of site-specific histone methylation in vitro and in vivo. Nat. Cell Biol..

[122] Khazaei S., Chen C.C.L., Andrade A.F., Kabir N., Azarafshar P., Morcos S.M., França J.A., Lopes M., Lund P.J., Danieau G. (2023). Single substitution in H3.3G34 alters DNMT3A recruitment to cause progressive neurodegeneration. Cell.

[123] Sankar A., Mohammad F., Sundaramurthy A.K., Wang H., Lerdrup M., Tatar T., Helin K. (2022). Histone editing elucidates the functional roles of H3K27 methylation and acetylation in mammals. Nat. Genet..

[124] Sarthy J.F., Meers M.P., Janssens D.H., Henikoff J.G., Feldman H., Paddison P.J., Lockwood C.M., Vitanza N.A., Olson J.M., Ahmad K. (2020). Histone deposition pathways determine the chromatin landscapes of H3.1 and H3.3 K27M oncohistones. eLife.

[125] Filipescu D., Müller S., Almouzni G. (2014). Histone H3 variants and their chaperones during development and disease: contributing to epigenetic control. Annu. Rev. Cell Dev. Biol..

[126] Athwal R.K., Walkiewicz M.P., Baek S., Fu S., Bui M., Camps J., Ried T., Sung M.H., Dalal Y. (2015). CENP-A nucleosomes localize to transcription factor hotspots and subtelomeric sites in human cancer cells. Epigenetics Chromatin.

[127] Nye J., Sturgill D., Athwal R., Dalal Y. (2018). HJURP antagonizes CENP-A mislocalization driven by the H3.3 chaperones HIRA and DAXX. PLoS One.

[128] Shrestha R.L., Ahn G.S., Staples M.I., Sathyan K.M., Karpova T.S., Foltz D.R., Basrai M.A. (2017). Mislocalization of centromeric histone H3 variant CENP-A contributes to chromosomal instability (CIN) in human cells. Oncotarget.

[129] Gomes A.P., Ilter D., Low V., Rosenzweig A., Shen Z.J., Schild T., Rivas M.A., Er E.E., McNally D.R., Mutvei A.P. (2019). Dynamic incorporation of histone H3 variants into chromatin is essential for acquisition of aggressive traits and metastatic colonization. Cancer Cell.

